# Chitosan nanoparticle-mediated delivery of miRNA-34a decreases prostate tumor growth in the bone and its expression induces non-canonical autophagy

**DOI:** 10.18632/oncotarget.4971

**Published:** 2015-07-22

**Authors:** Sanchaika Gaur, Yunfei Wen, Jian H. Song, Nila U. Parikh, Lingegowda S. Mangala, Alicia M. Blessing, Cristina Ivan, Sherry Y. Wu, Andreas Varkaris, Yan Shi, Gabriel Lopez-Berestein, Daniel E. Frigo, Anil K. Sood, Gary E. Gallick

**Affiliations:** ^1^ Department of Genitourinary Medical Oncology, David H. Koch Center for Applied Research of Genitourinary Cancers, The University of Texas, MD Anderson Cancer Center, Houston, TX, USA; ^2^ Program in Cancer Biology and Cancer Metastasis, The University of Texas Graduate School of Biomedical Sciences at Houston, TX, USA; ^3^ Department of Gynecologic Oncology and Reproductive Medicine, The University of Texas MD Anderson Cancer Center, Houston, TX, USA; ^4^ Center for RNA Interference and Non-Coding RNA, The University of Texas MD Anderson Cancer Center, Houston, TX, USA; ^5^ Center for Nuclear Receptors and Cell Signaling, Departments of Biology and Biochemistry, University of Houston, TX, USA; ^6^ Department of Experimental Therapeutics, The University of Texas MD Anderson Cancer Center, Houston, TX, USA; ^7^ Genomic Medicine Program, The Houston Methodist Research Institute, Houston, TX, USA; ^8^ Department of Cancer Biology, The University of Texas MD Anderson Cancer Center, Houston, TX, USA; ^9^ Department of Biomedical Sciences, Cedars Sinai Medical Center, Los Angeles, CA, USA

**Keywords:** prostate cancer, miR-34a, bone metastasis, apoptosis, autophagy

## Abstract

While several new therapies are FDA-approved for bone-metastatic prostate cancer (PCa), patient survival has only improved marginally. Here, we report that chitosan nanoparticle-mediated delivery of miR-34a, a tumor suppressive microRNA that downregulates multiple gene products involved in PCa progression and metastasis, inhibited prostate tumor growth and preserved bone integrity in a xenograft model representative of established PCa bone metastasis. Expression of miR-34a induced apoptosis in PCa cells, and, in accord with downregulation of targets associated with PCa growth, including MET and Axl and c-Myc, also induced a form of non-canonical autophagy that is independent of Beclin-1, ATG4, ATG5 and ATG7. MiR-34a-induced autophagy is anti-proliferative in prostate cancer cells, as blocking apoptosis still resulted in growth inhibition of tumor cells. Thus, combined effects of autophagy and apoptosis are responsible for miR-34a-mediated prostate tumor growth inhibition, and have translational impact, as this non-canonical form of autophagy is tumor inhibitory. Together, these results provide a new understanding of the biological effects of miR-34a and highlight the clinical potential for miR-34a delivery as a treatment for bone metastatic prostate cancer.

## INTRODUCTION

Prostate cancer (PCa) is the second leading cause of cancer-related deaths in men in the United States [1], with mortality due mainly to metastasis to the bone [2]. While several new therapeutic agents have been approved for the treatment of bone metastasis [3, 4], their ability to prolong life has been limited due to the rapid development of resistance [2, 5]. One approach to combat resistance is through combination therapies targeting several pathways in both the cancer cells and their tumor-promoting microenvironment. A promising strategy utilizing the same principle is miRNA-based therapeutics, since a single miRNA can have multiple targets in both the tumor cells themselves and the tumor microenvironment [6-9]. Restoring the expression of a tumor suppressive miRNA that is downregulated in cancer cells through nanoparticle-mediated delivery of mature miRNA mimics has been shown to decrease tumor growth in several pre-clinical models without toxic side effects [10-13].

We used an unbiased systems-based approach to identify miRNAs that are downregulated in prostate cancer with the goal of targeting multiple gene products that are overexpressed in this disease and that promote tumor progression and bone metastasis. This approach led to identifying miR-34a, a tumor suppressive miRNA that is downregulated in the advanced stages of prostate cancer. Importantly, miR-34a targets several gene products associated with advanced PCa, including c-Myc, MET and Axl in tumor cells [12, 14, 15] and Tgif2 in the bone microenvironment [16]. Additionally, previous studies have demonstrated that miR-34a delivery can inhibit the growth of PCa xenografts in immunodeficient mice [12, 15]. However, the complex biologic processes mediated by miR-34a that lead to tumor inhibition are not well described. Besides apoptosis, pathways that promote cell death include: autophagy-associated cell death [17], autosis [18], paraptosis [19], entosis [20], ferroptosis [21] and programmed necrosis [22], all of which can contribute to tumor inhibition. Among them, only autophagy has been described as both pro-tumorigenic by promoting survival under nutritional or chemotherapeutic stress and conversely, tumor suppressive, by promoting cancer cell death depending on the cellular context and different inducers of autophagy [17, 23-26]. These diametrically opposed biologic effects may be due, in part, to recent understanding that “canonical” and “non-canonical” autophagy pathways requiring different intermediates can elicit diverse effects in tumor cells [27, 28]. As decreased expression of the miR-34a targets, MET and Axl is associated with the induction of autophagy [29, 30], understanding the form of autophagy induced by miR-34a in prostate cancer is important for therapeutic opportunities for miR-34a replacement in bone metastatic prostate cancer.

The goal of this study was to determine whether miR-34a delivery inhibits prostate tumor growth in the bone and the mechanism of miR-34a-mediated tumor inhibition. Our results demonstrate that chitosan nanoparticle-mediated delivery of miR-34a decreases established prostate tumor growth in the bone and preserves bone integrity. Mechanistically, miR-34a induces apoptosis along with a form of autophagy that inhibits prostate cancer cell proliferation by a mechanism independent of expression of Beclin-1, ATG4, ATG5 and ATG7.

## RESULTS

### MiR-34a is downregulated in increasing stages of prostate cancer and in metastatic prostate cancer cell lines while its targets are overexpressed in metastatic PCa cells

**Figure 1:**
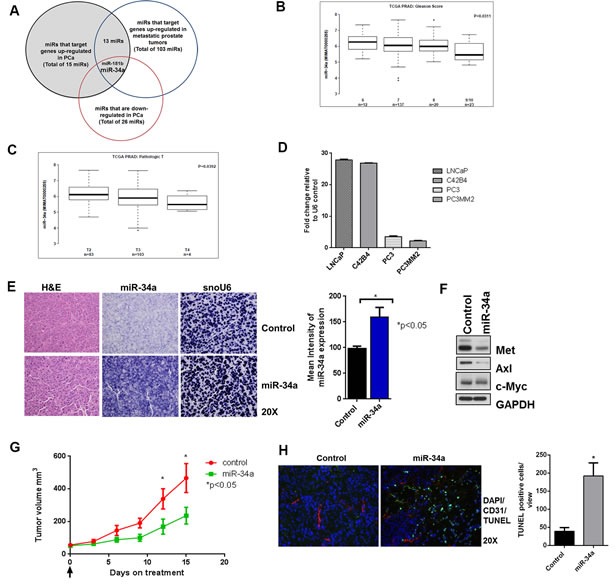
miR-34a is downregulated in prostate cancer and its delivery decreases sub-cutaneous tumor growth **A.** GEO datasets were analyzed for prostate cancer and a Venn diagram representing downregulated miRNAs in prostate cancer that target genes upregulated in prostate cancer and prostate cancer metastasis is shown. **B.** TCGA dataset was analyzed for miR-34a expression in high-grade prostate cancer with Gleason score (6-9/10) and **C.** for different pathologic T staging (T2-T4). **D.** miR-34a expression was measured by qPCR using TaqMan assay and compared with U6 endogenous control for LNCaP, C42B4, PC3 and PC3MM2. **E.** Formalin fixed paraffin embedded (FFPE) slides were stained with H&E or *in situ* hybridization was performed for miR-34a and endogenous control for small nuclear RNA U6 (snoU6) (left panel). The mean intensities for 10 areas from each slide at 10x magnification were measured with NIS Elements software (right panel) **F.** Western blots for MET, Axl and c-Myc from sub-cutaneous tumor treated with control or miRa-34a. **G.** Tumor volume of sub-cutaneous PC3MM2 xenografts was measured by calipers for control and miR-34a treated group (*n* = 6). **H.** A representative image is shown for control and miR-34a-treated tumors with TUNEL (green), nuclear DAPI (blue) and CD31 (red) staining (left panel). TUNEL-positive cells from 10 fields per tumor were quantified using ImageJ software and the mean and standard deviation is plotted (right panel).

To identify miRNAs that target at least seven or more genes frequently upregulated in prostate cancer relative to the normal prostate gland, we used GEO datasets (http://www.ncbi.nlm.nih.gov/gds, GSE6919) and then determined overlap with miRNAs that target genes upregulated in metastatic prostate cancer relative to primary cancer (GSE3325). Using this strategy, 13 miRNAs were identified. We next determined which of these 13 miRNAs are downregulated in PCa [31-35] and identified two miRNAs, miR-18b and miR-34a, that met these criteria (Figure 1A). We next compared miR-34a expression in the prostate cancer TCGA dataset (https://tcga-data.nci.nih.gov/tcga) with Gleason score (Figure 1B) and T stage (pathologic T) (Figure 1C), which demonstrates that miR-34a is downregulated in high-grade and advanced prostate cancer. To examine if a similar relationship occurred in human prostate cancer cell lines that could serve as a relevant, tractable experimental model, we quantified miR-34a expression levels by qPCR in LNCaP (which do not metastasize in immunocompromised mice), C42B4 (a LNCaP variant of low metastatic potential), PC3 (high metastatic potential) and PC3MM2 (selected for increased metastatic potential relative to parental PC3 cells). Expression of miR-34a was decreased by 8-fold in PC3 and by 12-fold in PC3MM2 compared to C42B4 and LNCaP cells (Figure 1D). This result is in agreement with the clinical data that miR-34a expression is inversely proportional to increasing grade and stages of PCa. Next, we examined the expression of selected miR-34a targets (MET, Axl and c-Myc) associated with increased metastatic potential of PCa cell lines. Each of these genes is increased at mRNA (Figure S1A) and protein (Figure S1B) levels in the highly metastatic PC3MM2 and PC3 cells. To determine whether increasing miR-34a expression leads to downregulation of these targets, we transiently transfected low miR-34a expressing PC3 and PC3MM2 cells with negative control (N.C.) miRNA or a miRNA-34a (miR-34a) mimic. Transfection of miR-34a decreased mRNA (Figure S1C) and protein (Figure S1D) levels of MET, Axl, and c-Myc in PC3 and PC3MM2 cells. Our data thus demonstrate that miR-34a levels are decreased in high metastatic PCa cells compared to low metastatic cells, and increasing miR-34a expression in high metastatic PCa cells simultaneously inhibited the expression of multiple targets that contribute to PCa progression and metastasis.

### Systemic miR-34a delivery by chitosan nanoparticles inhibits prostate tumor growth *in vivo*

We next tested whether miR-34a delivery affected tumor growth and whether all three targets could be downregulated by its delivery *in vivo*. For these studies, tumors were first grown subcutaneously as described in the materials and methods. Next, miR-34a was delivered to these tumors systemically via tail vein injections. To deliver miR-34a, we chose chitosan (CH) nanoparticles that are composed of biodegradable, naturally occurring polysaccharides with low immunogenicity and toxicity [36, 37]. We observed robust expression of miR-34a in tumors following delivery of miR-34a-CH as visualized by *in situ* hybridization (Figure 1E). Expression of miR-34a correlated with downregulation of MET, Axl and c-Myc as determined by immunoblotting (Figure 1F). Delivery of miR-34a decreased subcutaneous tumor growth (Figure 1G) and induced apoptosis as measured by an increase in TUNEL-positive cells (Figure 1H) in miR-34a treated tumors compared to control tumors. Collectively, these results demonstrate that nanoparticle-mediated delivery of miR-34a decreased the expression of its targets and tumor growth, as well as induced apoptosis in a subcutaneous model of prostate cancer.

### Effects of miR-34a delivery on PCa tumor growth in the bone

Since bone metastasis is the leading cause of death in PCa, our focus was on determining the effects of systemic miR-34a-CH delivery on established tumors in an intra-femoral model to represent treatment of PCa bone metastasis. To first determine whether chitosan could deliver small RNAs to the bone, we delivered Cy5.5-labeled siRNA through chitosan nanoparticles since the fluorescent signal from Cy5.5 can be detected by *ex vivo* imaging. PC3MM2-LG cells were injected in the femur of nude mice, and 10 days after tumor injection, unlabeled control or Cy5.5-labeled siRNA in chitosan nanoparticles were delivered intravenously. Fluorescence intensity was measured from harvested legs of animals sacrificed 3 days after siRNA delivery. An increase in Cy5.5-siRNA signal intensity was observed in the femur with tumor than in the femur without tumor (Figure S2) suggesting that siRNA delivered by chitosan nanoparticles is preferentially retained in the tumor growing inside the bone. Thus, chitosan nanoparticles were suitable for delivery of miR-34a to the bone.

**Figure 2:**
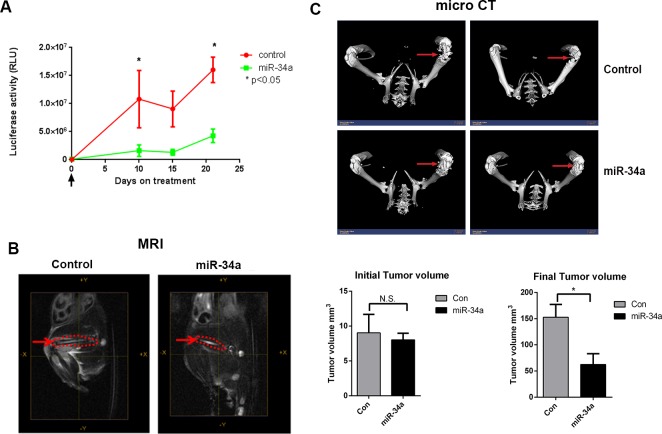
Systemic miR-34a delivery by chitosan nanoparticles decreases prostate tumor growth in bone in an intra-femoral model **A.** Bioluminescence activity to assess for intra-femur tumor growth was measured using an IVIS 200 for control and miR-34a groups (*n* = 5). **B.** Representative MRI images taken at the end of the experiment of the femurs (red dotted line) for control-CH and miR-34a-CH treated mice are shown. Tumor volume in the femur was measured before (left panel) and after (right panel) miR-34a delivery. **C.** Micro CT images for two control-CH and two miR-34a-CH treated mice. Red arrows indicate bone lesions. * denotes *p* < 0.05 as measured by Student's *t* test.

We next determined the effect of systemic miR-34a delivery on established tumors in the femur to best mimic treatment of bone metastasis. We injected PC3MM2-LG (transfected to express luciferase and GFP) cells into the femurs of nude mice and monitored tumor growth by bioluminescence activity and MRI. After ten days, when tumors were evident in the femurs (as demonstrated by MRI), mice were randomized and treated with either control-miRNA (scrambled sequence of negative control miRNA that does not interfere with known miRNA functions) or miR-34a chitosan nanoparticles every three days for three weeks through systemic administration. Delivery of miR-34a robustly decreased tumor growth relative to control group (measured by bioluminescence activity of PC3MM2-LG cells) (Figure 2A) and tumor volume (measured by MRI) (Figure 2B, right panel) of established prostate tumors in the bone. PC3MM2 cells cause lytic reactions in the bone. Importantly, miR-34a delivery led to a preservation of bone integrity as visualized by micro CT analysis (Figure 2C). Collectively, our results demonstrate that miR-34a's anti-tumor effects were superior in an intra-femoral PCa model compared to a sub-cutaneous model, suggesting that miR-34a may mediate tumor suppressive effects by targeting both the tumor as well as the bone microenvironment.

### MiR-34a inhibits metastatic properties in PC3 cells

To understand the mechanism of miR-34a-induced tumor inhibition, we expressed miR-34a in PC3 cells through transient transfection of miR-34a mimics and studied the biological effects. Expression of miR-34a decreased the ability of PC3 cells to migrate by 50% (Figure S3A), and the ability to invade by 75% (Figure S3B). Since miR-34a expression was maintained 96 hours post-transfection (data not shown), we determined the effects of increasing miR-34a expression on cell proliferation. Transfection of miR-34a decreased cell proliferation compared to N.C. (Figure S3C). We next performed cell cycle analysis using propidium iodide (PI) at various times after N.C. or miR-34a transfection. A 4-fold decrease in S-phase was observed beginning at 48 hours, which was maintained through 96 hours post-transfection (Figure S3D). After 72 hours, the sub G1 phase increased by 1.5 fold in miR-34a overexpressing cells, reaching a maximum of 2-fold at 96 hours relative to N.C. transfected cells (Figure S3D). These results demonstrate that miR-34a-mediated decreased cell proliferation is due, at least in part, to increased cell death. To determine what type(s) of cell death were occurring, we used a GFP-Certified® Apoptosis/Necrosis detection kit as described in the materials and methods. An increase in early (AnnVEnzoGold^+^/7AAD^−^) and late apoptotic (AnnVEnzoGold^+^/7AAD^+^) cell populations was observed at 72 and 96 hours post miR-34a transfection compared to N.C. transfected cells (Figure S3E). Collectively, our data demonstrate that increasing miR-34a expression has direct effects on tumorigenic and metastatic potential of PCa cells through inducing apoptosis, decreasing cell proliferation, and decreasing cell migration and invasion.

### MiR-34a induces autophagy

**Figure 3:**
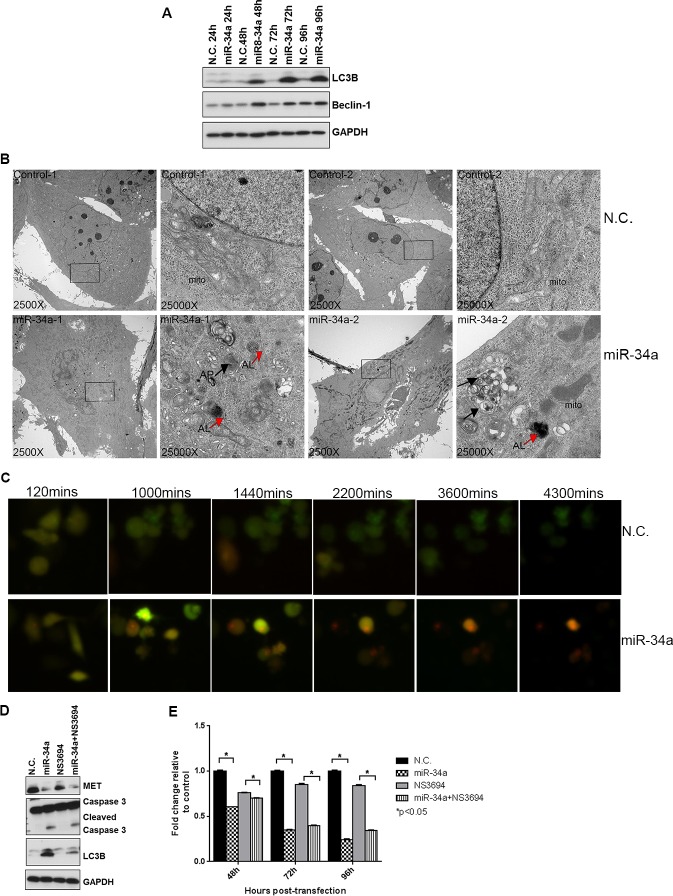
miR-34a induces autophagy in PC3 cells **A.** Western blots for LC3B, Beclin-1 and GAPDH from negative control (N.C.) and miR-34a transfected cells at indicated time points. **B.** Transmission electron microscopy (TEM) images at 2500X and 25000X magnification were captured for N.C. and miR-34a transfected PC3 cells at 72h. Black arrows indicate autophagosome (AP)-like and red arrows indicate autolysosome (AL)-like structures. **C.** Screen shots from time-lapse microscopy monitoring autophagic flux for PC3 cells labeled with GFP-RFP-tagged LC3 and transfected with either N.C. or miR-34a. **D.** Western blots for MET, cleaved caspase 3, LC3B and GAPDH from PC3 cells pre-treated with NS3694 (100nM) for 24 h, followed by N.C. or miR-34a transfection for 72 hours. **E.** Proliferation of cells pre-treated with NS3694 (100nM) for 24h, followed by N.C. or miR-34a transfection for indicated time points. * denotes *p* < 0.05 as measured by Student's *t* test.

Since downregulation of MET or Axl has been shown to induce both apoptosis and autophagy in different cancer types [29, 30], we asked whether miR-34a induces autophagy in addition to apoptosis in PCa cells. We first analyzed Beclin-1 expression in cells transfected with N.C. or miR-34a, as it is involved in vesicle nucleation and autophagosome formation [38, 39]. An increase in Beclin-1 protein, but not mRNA expression (data not shown), was observed at 48 hours in miR-34a transfected cells, and continued throughout the time course examined (Figure 3A). Next, we examined the conversion of LC3BI to LC3BII, a hallmark of autophagosome maturation [40]. Increased LC3BII levels were observed 48 hours after miR-34a transfection, and these increased levels were maintained at 72 and 96 hours post-transfection (Figure 3A).

To determine whether autophagic structures were formed in miR-34a transfected cells, transmission electron microscopy (TEM) was performed. As shown in Figure 3B, miR-34a transfection led to an accumulation of autophagosome (AP)-like structures (black arrows) as well as autolysosome (AL)-like structures (red arrows) that were not observed in N.C. cells. This result confirms that transfection of miR-34a in PC3 cells led to the accumulation of autophagosomes and autolysosomes. To monitor the autophagic flux induced by miR-34a in a real-time manner (indicative of the dynamic autophagic flux occurring from initiation to maturation of autophagosomes and fusion with lysosomes), cells pre-labeled with tandem monomeric RFP-GFP-tagged LC3 [41] and transfected with N.C. or miR-34a were visualized by time-lapse microscopy [40]. We observed that the signal from GFP+ and RFP+ vesicular structures in the N.C. and miR-34a transfected cells merged at 120 minutes (Figure 3C and Supporting Movies 1 and 2). However, at increasing times, miR-34a caused a sustained accumulation of RFP+ vesicular structures in the cytoplasm compared to the N.C. cells (Figure 3C, red cells; Supporting Movie 2 compared to Supporting Movie 1), indicative of accumulation of late-stage autophagic vesicles including fused autolyosomes, since the RFP signal is more stable than GFP in the acidic compartment of the lysosomes [40].

To quantitatively measure the population of acidic vesicular organelles (AVOs) that were accumulated in the cytoplasm during late-stage autophagy, cells transfected with N.C. or miR-34a for increasing times were stained with acridine orange and analyzed by flow cytometry as described in materials and methods. An increase in acridine orange-positive cells was observed in miR-34a-expressing cells at 48 hours with further increases noted at 72 and 96 hours (Figure S4A), mirroring the time frame observed for miR-34a-mediated increased LC3BII levels. Using confocal microscopy, we demonstrated that transfection of miR-34a for 72 hours increased the accumulation of acridine orange in the cytoplasm of cells (Figure S4B), further corroborating the FACS results. We next tested whether miR-34a led to degradation of p62/SQSTM1, a ubiquitin-binding scaffold protein that serves as link between autophagy process and protein degradation [42] in PC3 cells. Transfection with miR-34a decreased p62/SQSTM1 levels compared to control and N.C. transfected cells (Figure S4C). We further tested whether blocking autophagy by using hydroxychloroquine (HCQ) that prevents fusion of autophagosomes with lysosomes [43] affects miR-34a induced autophagy. HCQ prevented p62 degradation while miR-34a led to reduced levels of p62 in PC3 cells (Figure S4C). Pre-treatment with HCQ prevented miR-34a-mediated degradation of p62, which indicates that HCQ impedes miR-34a induced autophagy. Thus, the combined results demonstrate that miR-34a induces autophagy in PC3 cells.

To determine whether miR-34a-induced autophagy was inhibiting or promoting PC3 cell proliferation, we used the apoptosis inhibitor, NS3694 to inhibit the formation of the apoptosomes, followed by transfection of N.C. or miR-34a for 72 hours. The ability of NS3694 to block docetaxel-induced apoptosis (as determined by inhibition of caspase 3 activation) is shown in Figure S4D. Transfection with miR-34a decreased MET expression and increased LC3BII and cleaved caspase 3 levels even in the presence of the apoptosis inhibitor (Figure 3D). Further, NS3694 was unable to block miR-34a's potent anti-proliferative effects (Figure 3E). These results suggest that autophagy induced by miR-34a was associated with decreased proliferation.

We next determined whether miR-34a induced autophagy in other PCa cell lines. Transfection of miR-34a increased Beclin-1expression in PC3MM2 cells and LC3BII levels in both PC3MM2 and C42B4 cells (Figure S4E). These increases were similar to those observed in the PC3 cells (Figure 3A and Figure S4E). This result was supported by the increase in acridine orange-positive cells observed in both PC3MM2 and C42B4 cells (Figure S4F) following miR-34a transfection. These combined results indicate that miR-34a induces autophagy in several PCa cell lines.

### MiR-34a-induced autophagy is Beclin-1-independent

**Figure 4:**
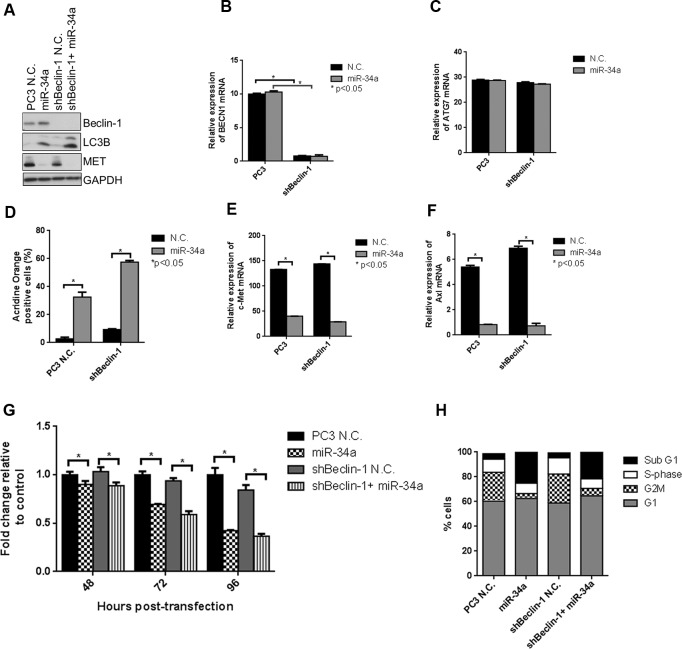
miR-34a induces autophagy, cell cycle arrest and decreases cell proliferation independently of Beclin-1 **A.** Western blot for Beclin-1, LC3B, MET and GAPDH for PC3 and shBeclin-1 cells with N.C. or miR-34a transfection. mRNA expression for **B.** Beclin-1 and **C.** ATG7 in PC3 and shBeclin-1 cells with N.C. or miR-34a transfection. **D.** Acridine orange-positive cells were quantified by Gallios FACS at 72 hours post- N.C. or miR-34a transfection. mRNA expression for **E.** c-Met and **F.** Axl in PC3 and shBeclin-1 cells with N.C. or miR-34a transfection. **G.** Proliferation of cells at indicated time points for PC3 and shBeclin-1 cells with N.C. or miR-34a transfection. **H.** Cell cycle analysis following propidium iodide staining and FACS for different cell cycle phases in PC3 and shBeclin-1 cells with 72h of N.C. or miR-34a transfection. * denotes *p* < 0.05 as measured by Student's t test.

Since miR-34a increased Beclin-1 protein expression, we tested whether miR-34a-induced autophagy is mediated through Beclin-1. A lentiviral shRNA was used to decrease expression of Beclin-1 in PC3 cells, which were then transfected with N.C. or miR-34a for 72 hours. Expression of Beclin-1 protein (Figure 4A) and mRNA (Figure 4B) was significantly decreased following lentiviral infection, while ATG7 mRNA expression was unaffected (Figure 4C). We next examined the effects of Beclin-1 knockdown on miR-34a-induced autophagy by determining LC3BII levels. Surprisingly, miR-34a expression increased LC3BII levels, independent of Beclin-1 knockdown (Figure 4A). This result was supported by the increase in acridine orange positive cells following miR-34a expression independent of Beclin-1 (Figure 4D). Further, miR-34a was still effective in inhibiting MET (Figure 4A and 4E) and Axl expression (Figure 4F). Consistent with our previous data, transfection of miR-34a in PC3 cells decreased proliferation compared to N.C. transfection (Figure 4G), while in shBeclin-1 cells, miR-34a also decreased cell proliferation compared to N.C. (Figure 4G). Additionally, cell cycle analysis demonstrated a 5-fold increase in the sub G1 fraction of cells following miR-34a expression in both control PC3 cells and in PC3 cells with Beclin-1 knockdown (Figure 4H). These data suggest that miR-34a-induced effects on autophagy, cell cycle and cell proliferation are mediated by a Beclin-1-independent mechanism.

### MiR-34a induces ATG5- and ATG7-independent autophagy

**Figure 5:**
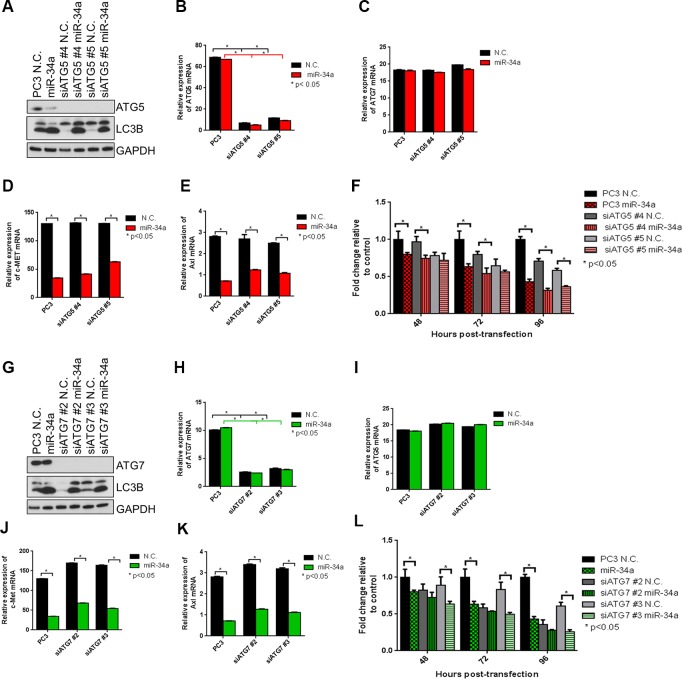
miR-34a induces autophagy and decreases cell proliferation independently of ATG5 and ATG7 **A.** Western blots for ATG5, LC3B and GAPDH in PC3 control cells and PC3 cells with two siATG5 sequences followed by N.C. and miR-34a transfection. mRNA expression for **B.** ATG5, **C.** ATG7, **D.** c-Met and **E.** Axl for PC3 control cells and PC3 cells with two siATG5 sequences with N.C. or miR-34a transfection after 72h as measured by qPCR. **F.** Proliferation of cells at indicated time points for PC3 control cells and PC3 cells with two siATG5 sequences followed by N.C. or miR-34a transfection. **G.** Western blots for ATG7, LC3B and GAPDH in PC3 control cells and PC3 cells with two siATG7 sequences followed by N.C. and miR-34a transfection. mRNA expression for **H.** ATG7, **I.** ATG5, **J.** c-Met and **K.** Axl for PC3 control cells and PC3 cells with two siATG7 sequences with N.C. or miR-34a transfection after 72h as measured by qPCR. **L.** Proliferation of cells at indicated time points for PC3 control cells and PC3 cells with two siATG7 sequences followed by N.C. or miR-34a transfection. * denotes *p* < 0.05 as measured by Student's *t* test.

Since several forms of non-canonical autophagy, including Atg5/Atg7-independent “alternative” macroautophagy have been reported [44, 45], we next determined whether miR-34a-induced autophagy was mediated through ATG5 and ATG7. In the canonical autophagy pathway, these ATGs are involved in autophagosome elongation and completion [46]. We used two siRNA sequences to robustly knockdown ATG5 and ATG7 expression in PC3 cells and then transfected them with N.C. or miR-34a. Success of knockdown was determined by immunoblotting and qPCR. ATG5 and ATG7 were reduced more than 90% at the protein (Figure 5A and Figure 5G) and mRNA (Figure 5B and Figure 5H) levels by corresponding siRNAs. Specificity of knockdown is indicated, as mRNA expression of ATG7 in siATG5 (Figure 5C) and ATG5 in siATG7 (Figure 5I) cells was unaffected. As expected, siATG5 and siATG7 decreased LC3II levels compared to N.C. (Figure 5A and 5G- lane 1 *vs*. lane 3 and lane 1 *vs*. 5) confirming that knocking down expression of these gene products inhibits basal autophagy. We then examined the effects of miR-34a on autophagy when either ATG5 or ATG7 were reduced by siRNA knockdown. As observed previously (Figure 3A), miR-34a expression increased LC3BII compared to N.C. (Figure 5A and 5G lane 1 *vs*. 2). Surprisingly, in the cells in which either ATG5 or ATG7 were reduced, miR-34a still increased LC3BII levels compared to N.C. (Figure 5A and 5G, compare lane 3 and 4 and lane 5 and 6). As expected, both c-Met (Figure 5D and 5J) and Axl (Figure 5E and 5K) mRNA levels were decreased following miR-34a transfection, regardless of ATG5 and ATG7 status. These results indicate that reduced expression of ATG5 or ATG7 did not block the autophagic flux induced by miR-34a, nor affected its downstream targets.

To determine miR-34a's effects on cell proliferation in PC3 cells and PC3 cells in which ATG5 or ATG7 was reduced by siRNAs, we used a fluorescent DNA dye to quantitate cell numbers as previously described [47]. Consistent with our previous data (Figure S3C), miR-34a transfection decreased proliferation compared to N.C. transfected cells (Figure 5F and 5L-column 1 *vs*. 2). Additionally, reduced ATG5 or ATG7 expression decreased both basal autophagy and cell proliferation compared to N.C. (Figure 5F and 5L-column 3 and 4 *vs*. column 1), consistent with previous reports [47]. However, miR-34a transfection further decreased proliferation in cells with reduced ATG5 (Figure 5F-column 3 *vs*. 4 and 5 *vs*. 6) and ATG7 (Figure 5L-column 3 *vs*. 4 and 5 *vs*. 6). This result suggests that ATG5 and ATG7 affect basal autophagy and cell proliferation, but not miR-34a-induced autophagy and its effect on cell proliferation.

To minimize any potential effects due to transient transfection, we created PC3 cells that stably expressed doxycycline-inducible shATG7. Upon addition of doxycycline, ATG7 protein (Figure S5A) and mRNA (Figure S5B) levels were decreased without affecting the levels of ATG5 (Figure S5C), confirming the inducible knockdown of ATG7. To examine the effects of shATG7 on miR-34a-induced autophagy, we determined LC3BII levels. Consistent with our results in PC3 cells transiently transfected with siATG7, an increase in LC3BII expression was still observed with miR-34a in both non-induced (Figure S5A lane 1 *vs*. 2) and doxycycline (Dox)-induced shATG7 (Figure S5A lane 3 *vs*. 4) cells. These results support that miR-34a effects on LC3B conversion are independent of ATG7. Similar to siATG7, miR-34a expression still decreased the mRNA levels of c-Met (Figure S5D) and Axl (Figure S5E) in both non-induced and shATG7 conditions. In addition, shATG7 decreased proliferation compared to non-induced cells (Figure S5F-column 1 *vs*. 3) while miR-34a further decreased proliferation in cells with reduced ATG7 expression (Figure S5F-column 3 *vs*. 4). Taken together, our data suggest that miR-34a induces autophagy independently of ATG5 and ATG7.

### MiR-34a overexpression effects autophagy independent of ATG4 knockdown

**Figure 6:**
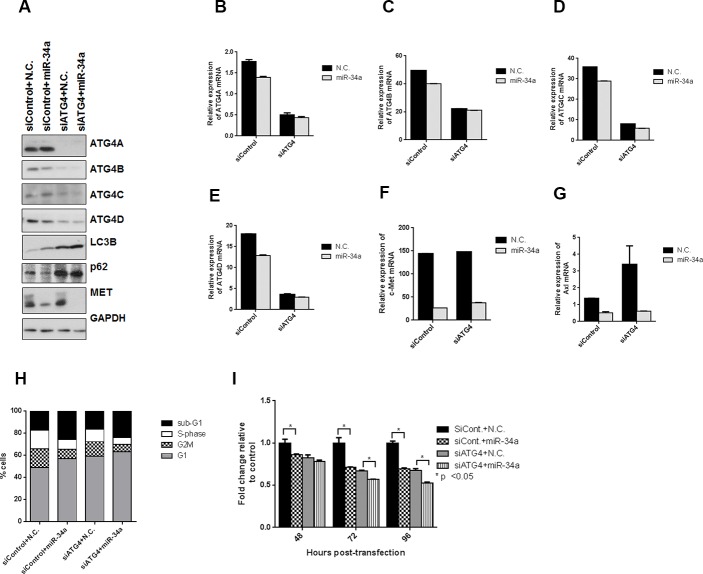
miR-34a induces autophagy independently of ATG4 All four isoforms of ATG4 were simultaneously knocked down in PC3 cells using pooled siRNAs. **A.** Western blots for ATG4A-D, LC3B, p62, MET and GAPDH. mRNA expression for **B.** ATG4A, **C.** ATG4B, **D.** ATG4C, **E.** ATG4D, **F.** c-Met and **G.** Axl for siControl and siATG4 cells with N.C. or miR-34a transfection after 72h as measured by qPCR. **H.** Cell cycle analysis following propidium iodide staining and FACS for different cell cycle phases in siControl and siATG4 cells with 72h of N.C. or miR-34a transfection **I.** Proliferation of cells at indicated time points for siControl and siATG4 cells with N.C. or miR-34a transfection. * denotes *p* < 0.05 as measured by Student's *t* test.

Since the intermediates involved in the downstream processing of LC3II (ATG7 and ATG5) did not affect autophagy induced by miR-34a, we examined whether ATG4, a cysteine protease involved both upstream in the conversion of pro-LC3 to LC3I and downstream in the recycling of LC3II to LC3I [48, 49], was required for miR-34a-induced autophagy. ATG4 has four isoforms, ATG4A, ATG4B, ATG4C and ATG4D with overlapping and distinct functions [48]. ATG4B was identified as a direct target of miR-34a in chronic myelogenous leukemia (CML), and the knockdown of ATG4B in CML led to impaired autophagic flux with an increase in LC3II levels and accumulation of p62 [50]. We thus determined whether miR-34a mediates its effects on autophagy through ATG4. To eliminate the redundant effects of ATG4 isoforms, we used pooled siRNA sequences to knock down all four isoforms simultaneously in PC3 cells and then transfected the cells with either control (N.C.) or miR-34a mimics. All isoforms of ATG4 were reduced with siRNA sequences, in protein (Figure 6A), as well as in mRNA (Figure 6B-6E) expression. Consistent with ATG4's role in LC3 recycling in the canonical autophagy pathway [49, 50], knockdown of the ATG4 isoforms increased LC3BII and led to p62 accumulation (Figure 6A). However, unlike the results in CML, miR-34a had only a minor effect on decreasing ATG4B protein expression (Figure 6A), suggesting that ATG4B is not a direct target of miR-34a in PC3 cells. Consistent with our above results, miR-34a expression in PC3 cells led to increased LC3BII levels (Figure 6A). Similarly, in line with the miR-34a induction of autophagy, p62 levels were decreased (Figure 6A). We next analyzed the effects of miR-34a transfection in siATG4 cells. We observed increased LC3BII levels and accumulation of p62, similar to what was observed with knockdown of ATG4 isoforms (Figure 6A). Targets of miR-34a are decreased at protein (Figure 6A) as well as mRNA levels (Figure 6F and 6G) with miR-34a expression in siATG4 cells similar to siControl cells. Cell cycle analysis demonstrated an increase in the sub G1 fraction of cells and a decrease in S-phase following miR-34a transfection in both control and siATG4 cells (Figure 6H). Correspondingly, there was a decrease in cell proliferation with miR-34a in siControl cells and miR-34a transfection further decreased proliferation in siATG4 cells (Figure 6I). These results suggest that while knockdown of ATG4 leads to impaired autophagy consistent with a previous report [50], miR-34a induces autophagy independent of ATG4 expression in prostate cancer cells.

## DISCUSSION

**Figure 7:**
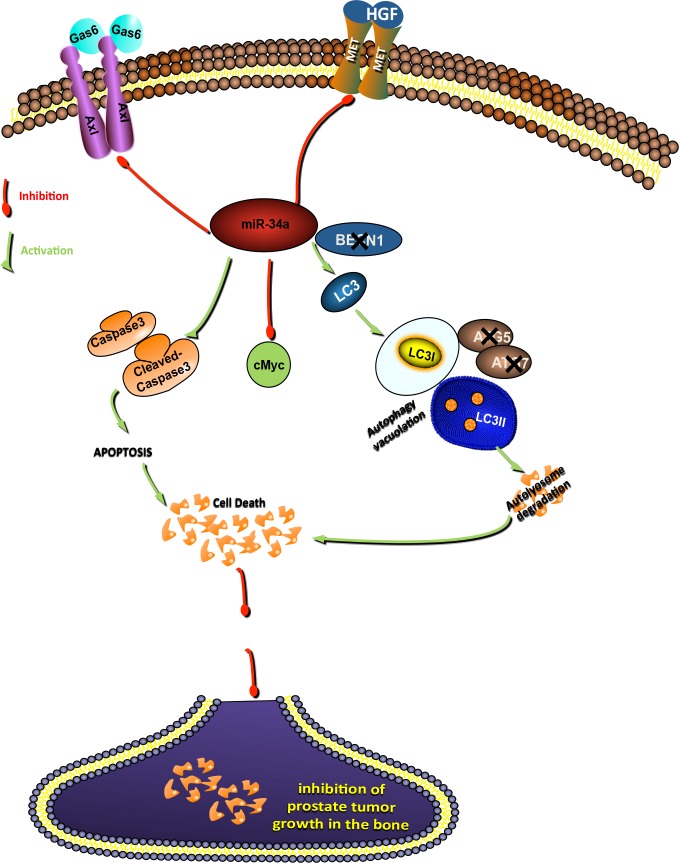
Schematic representation of signaling pathways by which miR-34a inhibits prostate cancer growth MiR-34a inhibits the expression of targets involved in promoting PCa progression and metastasis (c-Myc, Axl and MET). Inhibition of these targets activates both apoptosis and a non-canonical autophagy independent of Beclin-1, ATG4, ATG5 and ATG7. Both autophagy and apoptosis contribute to inhibition of prostate tumor growth.

We demonstrate that nanoparticle-mediated delivery of miR-34a inhibits the growth of bone-localized PCa. Overexpression of miRNA-34a induces a non-canonical form of autophagy in addition to apoptosis, inhibiting cell proliferation and promoting cell death in PCa cell lines of high metastatic potential. These data suggest that indirect induction of a non-canonical form of autophagy, through inhibition of targets such as MET and Axl contributes to inhibition of prostate tumor growth, as shown in the model in Figure 7. Our study highlights the tumor suppressive role of miR-34a-induced autophagy with clinical implications in the treatment of advanced bone metastatic PCa, the leading cause of death in prostate cancer. At present, the treatments available for this stage of the disease have limited success due to the rapid development of *de novo* or acquired resistance. Thus, therapeutic strategies that downregulate the expression of genes involved in multiple pathways that promote PCa growth in the bone may be more efficacious than targeted inhibitors with a more limited spectrum of targets, such as dasatinib and cabozantinib that have failed in phase 3 clinical trials [51]. MiRNA replacement therapy in advanced prostate cancers presents as an alternate treatment strategy for PCa bone metastasis due to their ability to target and downregulate oncogenic pathways in the cancer cells as well as in the bone microenvironment.

MiR-34a was chosen to test its therapeutic potential in a model for metastatic PCa growth in the bone based on: 1) its ability to target numerous gene products that promote PCa progression; 2) its downregulation in progressive stages of PCa; and 3) previous work demonstrating its delivery decreases tumor growth in orthotopic and subcutaneous PCa xenograft models [12, 15]. Our study demonstrates greater efficacy of miR-34a delivery in inhibiting tumor growth in the bone compared to a sub-cutaneous model implicating the anti-tumorigenic effects on both tumor cells and the bone microenvironment. Recently, Krzeszinski et al. [16] demonstrated that the miR-34a-mediated inhibition of breast and melanoma bone metastases was attributed exclusively to inhibiting osteoclast activity through downregulation of Tgif2, essential for osteoclast function [16]. Since osteoclast activation is implicated in bone remodeling and growth of PCa cells in the bone [52, 53], inhibition of Tgif2 and other miR-34a targets in the microenvironment may explain the more profound effects on tumor growth in the bone than were observed subcutaneously, and demonstrate the importance of targeting both the tumor cell and the tumor microenvironment. Thus, replacement of miR-34a with its ability to target and inhibit oncogenic gene products at both compartments presents as an alternate treatment strategy in metastatic prostate cancer.

Given the large number of potential miR-34a targets, some of which appear to be cell type-specific [54], and differences in potential “driver” oncogenes that promote metastatic growth among different cancer types, understanding the complexity of the biology due to miR-34a replacement therapy is critical to understand in which tumors delivery of this miRNA might lead to anti-tumor effects, and also the mechanisms by which this occurs. Downregulation of the miR-34a targets, MET and Axl have been shown to increase apoptosis as well as to induce autophagy [29, 30]. Accordingly, in prostate cancer cells, miR-34a led to delayed autophagy by classic criteria, including presence of autophagic structures as determined by TEM, immunoblotting for molecular markers of autophagy, and time-lapse microscopy to demonstrate autophagic flux. However, miR-34a-induced autophagy in these cells still occurred even when the well-studied intermediates of the canonical pathway (Beclin-1, ATG5, ATG7 and ATG4) were greatly decreased in expression by siRNAs/shRNAs (sufficiently decreased to block basal autophagy), suggesting a non-canonical form of this process. This non-canonical autophagy is anti-proliferative, as cell proliferation was decreased with miR-34a transfection even after inhibition of apoptosis.

Several examples of “non-canonical” autophagy have recently been reported. Similar to our results with Beclin-1 knockdown, Scarlatti, et al. [55] demonstrated that resveratrol induces Beclin-1-independent autophagy in breast cancer cells. Nishida et al. [45] reported an ATG5/ATG7-independent macroautophagy that did not increase LC3II levels. In contrast to this study, our results with miR-34a transfection increased LC3BII even in ATG5 and ATG7 knockdown cells, suggesting that other intermediates may be important in miR-34a-induced autophagy. Knockdown of ATG4B, a direct miR-34a target in CML impairs autophagy [50]. However, miR-34a had only a minor affect in decreasing ATG4B expression, suggesting that unlike CML cells [50], ATG4B is not likely a target of miR-34a in prostate cancer cells. Another example of cell type specificity of miR-34a targets is from the work of Liu et al. [56], who demonstrated that miR-34a inhibited autophagy under conditions of starvation or chemotherapy, leading to enhanced cell death in retinoblastoma cells. In that study, miR-34a altered autophagy by inhibiting HMGB1 expression [56]. In contrast, HMGB1 expression was not affected in prostate cancer cells by miR-34a (data not shown). Thus, miR-34a's effects on autophagy may differ depending on the conditions by which autophagy is induced and downregulation of different targets in different tissues (such as MET and Axl in prostate cancer cells). As the “forms” of autophagy are ever increasing, we speculate that different inducers mediate the autophagy process through different signal transduction pathways, which may account for the varied biological effects in different tumor types observed by miR-34a replacement, with critical implications as to whether autophagy is tumor promoting or suppressive or directly leads to apoptosis.

In conclusion, our study highlights the role of miR-34a in inducing autophagy along with apoptosis. Our study has therapeutic implications in the clinic, and emphasizes the need to understand the direct inducers and intermediates that lead to different forms of autophagy, so that predictions can be made as to whether this biologic process will function as an oncogenic process or as a tumor suppressive process.

## MATERIALS AND METHODS

### miRNA transfection

Transient transfections were performed using Lipofectamine 2000 transfection reagent (Invitrogen, Carlsbad, CA) for 24 hours, or 48, 72 or 96 hours for time course experiments. Briefly, 100,000 or 200,000 cells were placed in a 6-well plate in growth media without antibiotics 24 hours prior to transfection. Cells were then transfected with either negative control (N.C.) miRNA or miR-34a mimics (Ambion, Austin, TX) at a final concentration of 30 nM, according to the manufacturer's instructions.

### RNA isolation and quantitative polymerase chain reaction (qPCR)

Total RNA was isolated from cell lines using the mirVana kit (Ambion, Austin, TX) according to the manufacturer's instructions and 10 ng of total RNA was reverse transcribed using the TaqMan miRNA reverse transcription kit (Applied Biosystems, Foster City, CA). Quantitative PCR (qPCR) was performed on the Agilent 3000P system using the human miR-34a and U6 miRNA TaqMan expression assays (Applied Biosystems). Relative miR-34a expression was determined using the gene comparative C_T_ method. For gene expression analysis, 200 ng of total RNA was reverse transcribed using ThermoScript^TM^ RT-PCR system for first strand cDNA synthesis (Invitrogen, Carlsbad, CA) according to the manufacturer's instructions. Gene expression was then determined by qPCR using the KiCq Start SYBR Green kit (Sigma, St. Louis, MO). The primers sequences used for gene expression SYBR Green qPCR are listed in Supplemental Table 1.

### Immunoblotting

Protein lysates were prepared using RIPA B lysis buffer (150 mM NaCl, 20 mM sodium phosphate buffer, 5 mM ethylenediaminetetraacetic acid, and 1% Triton X-100, pH 7.4) along with 1 tablet of protease and phosphatase inhibitor cocktail (Roche, Indianapolis, IN). For *in vivo* samples, tumor sections were cut and homogenized by magnetic beads in RIPA A lysis buffer (1% Triton X-100, 0.1% SDS, 0.5% sodium deoxycholate, 150mM NaCl, 5mM EDTA, 5mM sodium pyrophosphate, 20mM sodium phosphate buffer, pH 7.4) along with 1 tablet of protease and phosphatase inhibitor cocktail). Total protein lysates (15 or 30 μg) were loaded onto an 8% or 12% polyacrylamide gel, which were then transferred to a polyvinylidene difluoride (PVDF) membrane and blocked with 5% milk in tris-buffered saline with Tween-20. Membranes were probed with specific primary antibodies: c-Met (C12), c-Myc (Santa Cruz Biotechnology, Santa Cruz, CA), Axl, cleaved caspase 3, LC3B, p62/SQSTM1, Beclin-1, ATG5 and ATG7 (Cell Signaling, Danvers, MA), GAPDH (Millipore, Temacula, CA), and horseradish peroxidase-conjugated secondary goat anti-mouse or goat ant-rabbit antibody (Bio-Rad, Hercules, CA).

### Transmission electron microscopy (TEM)

Samples fixed with a solution containing 3% glutaraldehyde plus 2% paraformaldehyde in 0.1 M cacodylate buffer, pH 7.3 were washed in 0.1 M cacodylate buffer and treated with 0.1% Millipore-filtered buffered tannic acid, post-fixed with 1% buffered osmium tetroxide for 30 min, and stained en bloc with 1% Millipore-filtered uranyl acetate. The samples were washed several times in water, then dehydrated in increasing concentrations of ethanol, infiltrated, and embedded in LX-112 medium. The samples were polymerized in a 60°C oven for 2 days. Ultrathin sections were cut in a Leica Ultracut microtome (Leica, Deerfield, IL), stained with uranyl acetate and lead citrate in a Leica EM Stainer, and examined in a JEM 1010 transmission electron microscope (JEOL, USA, Inc., Peabody, MA) at an accelerating voltage of 80 kV. Digital images were obtained using AMT Imaging System (Advanced Microscopy Techniques Corp, Danvers, MA).

### siRNA and shRNA transfection

The sequences of ATG5 and ATG7 chemical siRNAs were purchased from Life Technologies. Validated siRNAs for four isoforms of ATG4 (ATG4A, 4B, 4C and 4D) were purchased from Sigma. PC3 cells were transfected with 100 nM of siATG5 or siATG7 or 30 nM each of siATG4 sequences using DharmaFECT1 (GE Healthcare, Lafayette, CO) transfection reagent and 24 hours later, siATG5, siATG7 or siATG4 cells were transfected with N.C. or miR-34a using Lipofectamine 2000. Lentiviral shRNA constructs for Beclin-1 were provided by The University of Texas MD Anderson Cancer Center (UTMDACC) shRNA and ORFeome core facility. GIPZ lentiviral shBeclin-1 (GE Healthcare) constructs were packaged in lentivirus and PC3 cells were transduced with the concentrated viral titer in growth media containing 8 μg/ml polybrene and GFP+ cells were sorted by BD FACS Aria II following which they were transfected with either N.C. or miR-34a mimics for 72 hours.

### Animal studies

One million PC3MM2 cells were injected sub-cutaneously into nude mice. One week after cell injection, mice were randomized and divided into control group or miR-34a treatment group. The control group received negative control (N.C.) miRNA (scrambled sequence of negative control miRNA tested and validated to confirm that it does not interfere with known miRNA functions) encapsulated in chitosan nanoparticles (N.C.-CH) while the miR-34a group received miR-34a mimic encapsulated in chitosan nanoparticles (miR-34a-CH). Chitosan (CH) nanoparticles complexed with N.C. or miR-34a were prepared as described previously [57]. Briefly, CH solution was obtained by dissolving CH in 0.25% acetic acid and nanoparticles were spontaneously generated by the addition of TPP (0.25% w/v) and either N.C. or miR-34a at concentration of 1μg/μL under constant stirring at room temperature. After incubating at 4°C for 40 min, N.C.-CH or miR-34a-CH nanoparticles were collected by centrifugation at 14,000 rpm for 40 minutes at 4°C. The pellet was washed 3 times to remove unbound chemicals or N.C./miR-34a and N.C.-CH and miR-34a-CH nanoparticles were dissolved in water. 5μg/100μL of nanoparticles were delivered through tail vein injection per mouse, every three days for two weeks. Tumor volume as measured by caliper instrument was recorded. After two weeks of treatment, the animals were sacrificed and tumors harvested for protein, *in situ* hybridization (ISH) and immunofluorescence (IF). For intra-femur experiment, 1×10^6^ PC3MM2-LG cells labeled with luciferase and GFP were injected in the femur of the mice. Ten days after cell injection, mice were randomized into two groups- control and miR-34a and N.C.-CH or miR-34a-CH nanoparticles were delivered (5μg/100μL of nanoparticles) respectively via tail-vein injection every three days for three weeks. Tumor growth was monitored through bioluminescence imaging (IVIS 200) and tumor volume was measured before the start of treatment and at the end of treatment by magnetic resonance imaging (MRI) (Bruker 4.7T). Micro CT imaging was performed to visualize bone integrity at the end of the experiment on the Explore Locus RS pre-clinical *in vivo* scanner (GE Medical Systems, London Ontario).

### Statistics

Student's *t* tests were used for all statistical comparisons and a *p* value of <0.05 was considered statistically significant. Statistical analyses were performed using the GraphPad Prism software.

## SUPPLEMENTAL MATERIALS AND METHODS TABLE, MOVIES AND FIGURES


