# Supplementary material for: Chitosan nanoparticle-mediated delivery of miRNA-34a decreases prostate tumor growth in the bone and its expression induces non-canonical autophagy
Source: Oncotarget. 2015 Jul 22;6(30):29161–77. doi: 10.18632/oncotarget.4971 (PMC4745718; doi:10.18632/oncotarget.4971)
Supplement: Supplementary file 1 [file oncotarget-06-29161-s001.pdf]

Chitosan nanoparticle-mediated delivery of miRNA-34a decreases prostate tumor growth in the bone and its expression induces non-canonical autophagy

Supplemental Materials and Methods

Cell lines and media

PC3 and PC3MM2 cells were a gift from Dr. Isiah J. Fidler at The University of Texas MD Anderson Cancer Center (UTMDACC). LNCaP cells were purchased from American Type Culture Collection, and C42B4 and PC3MM2-LG (luciferase-GFP labeled) cells were a gift from Dr. Sue Hwa Lin at UTMDACC. PC3 cells with doxycycline-inducible shRNA knockdown of ATG7 (PC3 shATG7 no Dox/+Dox) were created and validated by Dr. Daniel E. Frigo's laboratory as previously described [44]. PC3 and PC3MM2 cells were maintained in 50% Dulbecco's Modified Eagle Medium / 50% F-12 (DMEM/F12, Sigma, St. Louis, MO) supplemented with 10% fetal bovine serum (Sigma, St. Louis, MO) and 1% penicillin/streptomycin (Hyclone, Logan, UT). LNCaP, and C42B4 cells were cultured in RPMI-1640 media (Sigma, St. Louis, MO) supplemented with 10% fetal bovine serum (FBS), sodium pyruvate, non-essential amino acids, Modified Eagle Medium vitamin solution (Invitrogen), and 1% penicillin/streptomycin. In the PC3 shATG7 inducible cells, 800 ng/ml of doxycycline was used to induce the knockdown of ATG7.

Bioinformatics analysis

Prostate cancer datasets with gene expression information were accessed from GEO datasets (http://www.ncbi.nlm.nih.gov/gds, GSE3325 and GSE6919). MiRNA target analysis was performed using miRWalk (http://www.umm.uniheidelberg.de/apps/zmf/mirwalk/). A gene product (mRNA) was

considered to be targeted by a given miRNA when the miRNA-mRNA relationship held true in at least 4 different target prediction algorithms. From the above datasets, we identified mRNAs that were commonly upregulated in prostate cancer compared to normal prostate and mRNAs upregulated in metastasis compared to primary cancer. The list of miRNAs targeting seven or more of the genes upregulated (5-fold increase) in prostate cancer was intersected with a list of miRNAs targeting genes upregulated (10-fold increase) in metastasis. Concurrently, the miRNAs that were downregulated in PCa from literature search were generated. MiRNAs that target genes that are upregulated in cancer or cancer metastasis and at the same time downregulated in cancer were included for further analysis. We also downloaded publically available data from the TCGA (https://tcga-data.nci.nih.gov/tcga/) for patients with prostate adenocarcinoma (PRAD). These analyses were performed in an R statistical environment (version 3.0.1) (http:///www.r-project.org/). The tests were two-sided and considered statistically significant when $p < 0.05$. The expression of miR-34a was obtained from the Illumina mirnaseq Level_3 data set. We derived from the "isoform_quantification" file the "reads_per_million_miRNA_mapped" values for miR-34a. To find the relationship between miR-34a expression and T staging (pathologic T), and the relationship between miR-34a and the Gleason score, we first employed a Shapiro-Wilk test to verify if the data followed a normal distribution. Accordingly, an ANOVA test, and the nonparametric Kruskal-Wallis test were applied to assess the relationship between miR-34a and the pathologic T, and between miR-34a and the Gleason score respectively. A box-and-whisker plot (Box plot represents first (lower bound) and third (upper bound)

quartiles, whiskers represent 1.5 times the interquartile range) was used to visualize the data (log2 (x+1)).

Migration and invasion assay:

Migration and invasion assays were performed using migration (catalog #354578) and invasion (catalog #354480) assay inserts (BD Biosciences, Bedford, MA). The number of migrated or invaded cells for 5 fields for each insert was counted under a bright-field microscope and plotted as the number of cells migrated or invaded per field. Each experiment was performed in triplicate.

Cell Proliferation assay:

For measuring cell viability, The CellTiter 96® AQueous Non-Radioactive Cell Proliferation Assay kit (Promega, Madison, WI) was used according to the manufacturer's instructions. FluoReporter® Blue Fluorometric dsDNA Quantitation kit (Life Technologies) containing Hoechst 33258 was used according to the manufacturer's instructions for cell proliferation analysis. Briefly, 2000 N.C. or miR-34a transfected cells -/+ siATG5, -/+siATG7, -/+ siATG4, -/+shATG7 or -/+shBeclin-1 were seeded in a 96-well plate and fluorescence (Ex/Em: 360nm/460nm) was measured and plotted as fold change relative to control (N.C.).

Inhibition of autophagy and apoptosis

Hydroxychloroquine (HCQ) was purchased from Sigma and used at concentration of 1nM. PC3 cells were pre-treated with HCQ for 24h prior to miR-34a transfection and cell

lysates were prepared. NS3694 was purchased from Sigma and 24h before N.C. or miRNA transfection, 100 nM of NS3694 was added to PC3 cells and following 72 hours of transfection, cell lysates were prepared for western blots. Proliferation of cells pre-treated with NS3694 and then transfected with N.C. or miR-34a was performed as described above.

Flow cytometry

Propidium iodide (PI) and RNAse A (Sigma) solutions were prepared according to the manufacturer's instructions. For cell cycle analysis, cells transfected with N.C. or miR-34a for different time points were fixed in 70% ethanol overnight. Fixed cells were then resuspended in 50 µg/ml PI solution in PBS with 0.2 mg/ml DNAase-free RNase A and incubated in a tissue culture incubator at 37°C/5% CO_2 for 30 minutes and analyzed by FACS Gallios. Singlet cell population was gated to exclude cell aggregates and percentage of cells in sub G1, G1, S and G2/M phase were recorded. GFP-Certified® Apoptosis/Necrosis detection kit (Enzo Life Sciences, Farmingdale, NY) was used for detection of early and late-stage apoptotic as well as necrotic cells. An AnnexinV-EnzoGold (enhanced Cyanine-3) (Ex/Em: 550/570nm) conjugate was used for detection of early stage apoptotic cells in the FL2 channel and Necrosis Detection Reagent, a proprietary dye similar to the red-emitting dye 7-AAD (Ex/Em: 546/647nm), was used for late apoptosis and necrosis detection in FL3 channel by FACS Gallios. Acridine Orange (Life Technologies) was used to measure acidic vesicular structures. Briefly, cells transfected with N.C. or miR-34a for different time points were incubated with 1µg/ml acridine orange (AO) for 30 min in the dark. In AO-stained cells, the cytoplasm or

nucleolus fluoresces bright green and dim red, whereas acidic compartments fluoresce bright red. Green (510–530 nm) and red (>650 nm) fluorescence emissions from 10,000 cells illuminated with blue (488 nm) excitation light were measured with a FACS Gallios.

siRNA and shRNA transfection

The sequences of ATG5 and ATG7 chemical siRNAs were purchased from Life Technologies. Validated siRNAs for four isoforms of ATG4 (ATG4A, 4B, 4C and 4D) were purchased from Sigma. PC3 cells were transfected with 100 nM of siATG5 or siATG7 or 30 nM each of siATG4 sequences using DharmaFECT1 (GE Healthcare, Lafayette, CO) transfection reagent and 24 hours later, siATG5, siATG7 or siATG4 cells were transfected with N.C. or miR-34a using Lipofectamine 2000. Lentiviral shRNA constructs for Beclin-1 were provided by The University of Texas MD Anderson Cancer Center (UTMDACC) shRNA and ORFeome core facility. GIPZ lentiviral shBeclin-1 (GE Healthcare) constructs were packaged in lentivirus and PC3 cells were transduced with the concentrated viral titer in growth media containing 8 μg/ml polybrene and GFP+ cells were sorted by BD FACS Aria II following which they were transfected with either N.C. or miR-34a mimics for 72 hours.

Autophagic flux assay

pGFP-RFP-LC3 plasmid was transfected into PC3 cells, and then cells were transfected with N.C. or miR-34a and plated on a 24-well plate. The GFP and RFP positive cells were visualized with a fluorescent microscope (Nikon) for time-lapse analysis. Each experiment was repeated at least three times, and representative images from control

(N.C) or miR-34a transfected PC3 cells were identified and combined in Image J software to generate autophagy flux videos.

Confocal Imaging

PC3 cells grown in 4-well chamber slide were transfected with N.C. or miR-34a for 72 hours and incubated with 1ug/ml acridine orange for 30 min in the dark. The cells were then fixed with 4% paraformaldehyde and sucrose solution and mounted. Fixed cells were then imaged under a confocal microscope at 20X magnification.

Immunofluorescence (IF) and in situ hybridization (ISH)

For TUNEL staining, DeadEnd colorimetric TUN-EL system (Promega) was used according to manufacturer's instructions. Briefly, formalin fixed paraffin embedded (FFPE) slides were deparaffinized and antigen retrieval was performed with 1X Dako Antigen Retrieval buffer. Slides were then washed with PBS, blocked with 3% H_2O_2 in methanol for peroxidase blocking, and in 4% fish gelatin for protein blocking. The slides were incubated with 4% paraformaldehyde in PBS for 10 minutes at room temperature (RT), washed with PBS, incubated in 0.2% TritonX-100 in PBS for 15 min at RT, washed with PBS and incubated with Equilibration buffer (Promega) for 10 min at RT. TUNEL incubation buffer (Promega) was added to each slide for 1 hour at 37°C in the dark. Slides were washed in 2X SSC (Promega) and counterstained with Hoechst mounting media (Life Technologies) and visualized under fluorescence microscope. DIG-labeled probes for miR-34a and U6 endogenous control were purchased from

Exiqon (Woburn, MA) and *in situ* hybridization (ISH) was performed on tumor sections according to the manufacturer's instructions by the Center for RNA Interference and non-coding RNA at UTMDACC. IF and ISH images were quantified by ImageJ and NIS software.

Cy5.5 delivery

Chitosan (CH) nanoparticles complexed with control or Cy5.5-siRNA were prepared as described previously [57]. Briefly, a CH solution was obtained by dissolving in 0.25% acetic acid and nanoparticles were spontaneously generated by the addition of TPP (0.25% w/v) and either control or Cy5.5-siRNA at a concentration of 1μg/μL to CH solution under constant stirring at room temperature. One million PC3MM2-LG cells were injected in the femur of the mice. Ten days after cell injection, mice received control or Cy5.5-CH nanoparticles (5μg/100μL of nanoparticles) via tail-vein injection. Three days after nanoparticle injection, mice were sacrificed; femurs were harvested and imaged with IVIS 200.

Supplemental Table 1: List of Primer Sequences

mRNA	ID	Primer sequence
Axl	Axl-F	5’-CGCAGGAGAAAGAGGATGTC-3’
	Axl-R	5’-ACCTACTCTGGCTCCAGGATG-3’
c-Met	Met-F	5’-CAGATGTGTGGTCCTTTG-3’
	Met-R	5’-ATTCGGGTTGTAGGAGTCT-3’
c-Myc	Myc-F	5’-TCAAGAGGTGCCACGTCTCC-3’
	Myc-R	5’-TCTTGGCAGCAGGATAGTCCTT-3’
ATG5	ATG5-F	5’-GAGTAGGTTTGGCTTTGGTTGA-3’
	ATG5-R	5’-CGTCCAAACCACACATCTCG-3’
ATG7	ATG7-F	5’-GCATCCAGAAGGGGGCTATG-3’
	ATG7-R	5’-AGGCTGACGGGAAGGACAT-3’
BECN1	BECN1-F	5’-GCGATGGTAGTTCTGGAGGC-3’
	BECN1-R	5’-AGACCCTTCCATCCCTCAGC-3’
ATG4A	ATG4A-F	5’-CTTCAAACCAGAGTGACGAGC-3’
	ATG4A-R	5’-CAGGCAATGGAAAGTCTGGTC-3’
ATG4B	ATG4B-F	5’-TCGCTGTGGGGTTTTTCTGT-3’
	ATG4B-R	5’-CACCTCCAAGCAGAGACAGC-3’
ATG4C	ATG4C-F	5’-TGTGTGGGTATTATTGGTGGC-3’
	ATG4C-R	5’-GGGCAGTGGAATGTCTCAAG-3’
ATG4D	ATG4D-F	5’-GGGCGAGGGTGACATACAG-3’
	ATG4D-R	5’-ACAGTCCGAGGTCAGGCA-3’
18s rRNA	18S-F	5’-GTAACCCGTTGAACCCCATT-3’
	18S-R	5’-CCATCCAATCGGTAGTAGCG-3’

Supplemental Movie Legends:

Supplemental Movie 1 control (NC): PC3 cells were plated on an optical 24-well plate and transfected with a GFP-RFP tagged LC3 plasmid followed by transfection with negative control (N.C.) mimic. For time-lapse, live video microscopy was performed in the RFP and GFP channel with an Olympus fluorescent microscope with 20-minute time interval for 72 hours. The movies were analyzed and time stamped using Image JPro software.

Supplemental Movie 2 miR-34a: PC3 cells were plated on an optical 24-well plate and transfected with a GFP-RFP tagged LC3 plasmid followed by transfection with miR-34a mimics. For time-lapse, live video microscopy was performed in the RFP and GFP channel with an Olympus fluorescent microscope with 20-minute time interval for 72 hours. The movies were analyzed and time stamped using Image JPro software.

Figure S1

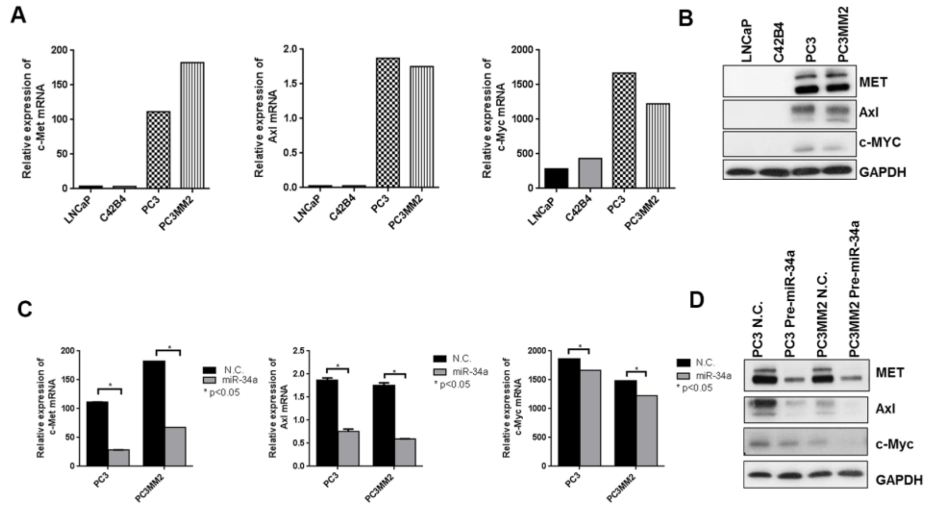

Figure S1: miR-34a targets are overexpressed in metastatic PCa cell lines. (**A**) mRNA expression of c-Met, Axl and c-Myc as measured by qPCR and normalized to 18S control. (**B**) Western blots for MET, Axl and c-Myc. (**C**) mRNA expression of c-Met, Axl and c-Myc after N.C. or miR-34a transfection in PC3 and PC3MM2 cells. (**D**) Western blots for miR-34a targets (MET, Axl and c-Myc) after N.C. or miR-34a transfection. * denotes p value <0.05 as measured by student's t test.

Figure S2

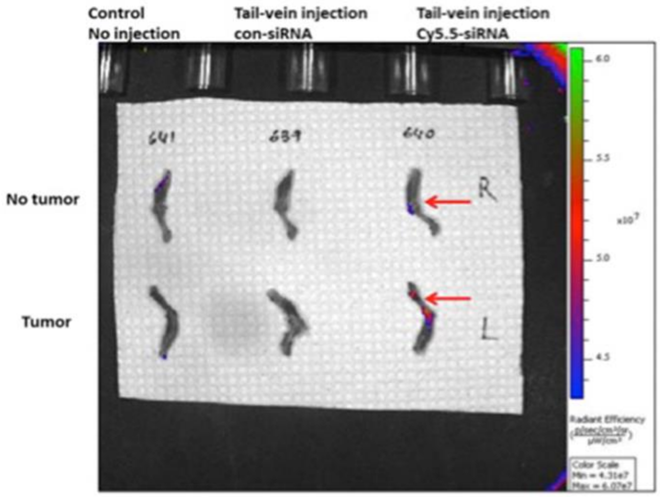

Figure S2: Chitosan nanoparticles deliver Cy5.5-siRNA to the femur. Control or Cy5.5-labeled siRNAs were encapsulated in chitosan nanoparticles and injected intravenously and imaged with IVIS200. Both femurs (with and without tumor growing) were harvested and subjected to ex vivo imaging. Red arrow indicates fluorescent signal from Cy5.5-siRNA from the femurs.

Figure S3

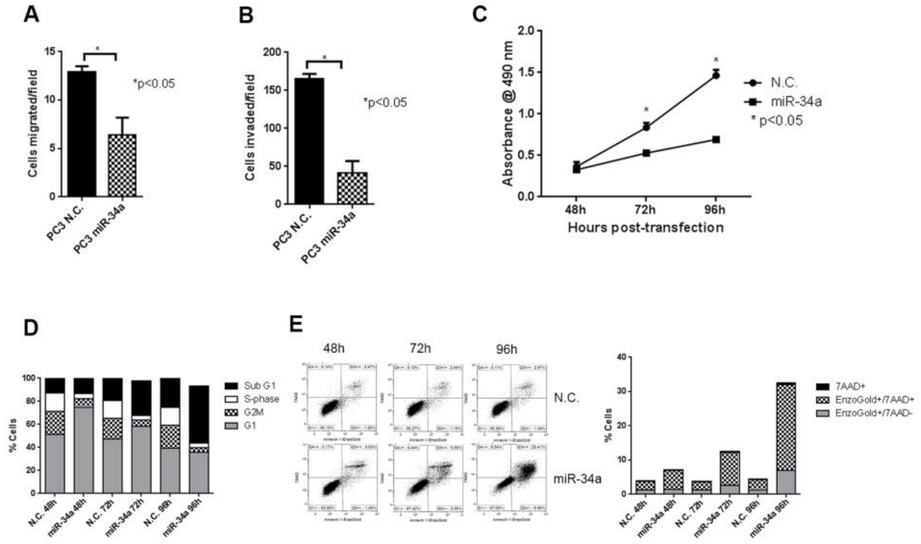

Figure S3 miR-34a inhibits metastatic properties of PC3 cells *in vitro*. (**A**) Migratory and (**B**) invasive ability of PC3 was measured after N.C. or miR-34a transfection by using modified Boyden chamber inserts in serum free media. Cells that migrated or invaded the matrigel layer were stained and quantified. (**C**) MTS assay was performed on N.C. or miR-34a transfected for indicated time points and mean absorbance at 490 nm from triplicate wells was determined. (**D**) Cell cycle analysis following propidium iodide staining and FACS for different cell cycle phases in N.C. or miR-34a transfected cells were quantified at indicated time points. (**E**) Apoptotic cells and necrotic cells in N.C. or

miR-34a transfected cells at indicated time points were analyzed by Gallios FACS (*left panel*) using GFP-Certified® Apoptosis/Necrosis detection kit and quantified (*right panel*). * denotes p value <0.05 as measured by student's t test.

Figure S4

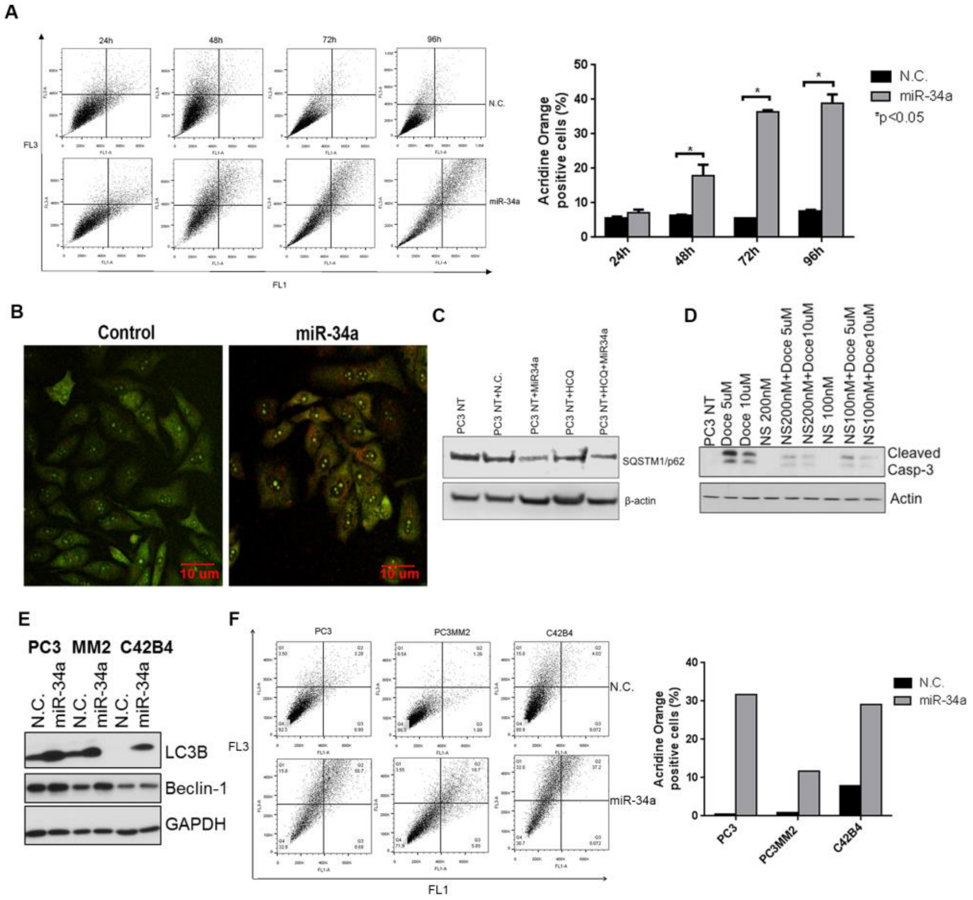

Figure S4: miR-34a induces autophagy in PCa cancer cell lines. (**A**) Acridine orange staining in PC3 cells transfected with N.C. or miR-34a for indicated time points was analyzed by Gallios FACS (*left panel*) and quantified (*right panel*). (**B**) Confocal images captured at 20X magnification of acridine orange stained PC3 cells transfected with N.C. or miR-34a for 72 hours. (**C**) Western blot for p62/SQSTM1 in PC3 cells treated with N.C, miR-34a, and hydroxychloroquine (HCQ), or combination of HCQ+miR-34a. (**D**) Western blots for cleaved caspase 3 in PC3 cells treated with docetaxel and combination of docetaxel with NS3694. (**E**)Western blots for LC3B, Beclin-1 and GAPDH after 72

hours of transfection with N.C. or miR-34a on lysates from different PCa cell lines. (**F**) Acridine orange staining in PCa cell lines (PC3, PC3MM2, and C42B4) transfected with N.C. or miR-34a for 72 hours was analyzed by Gallios FACS (*left panel*) and quantified (*right panel*). * denotes p value <0.05 as measured by student's t test.

Figure S5

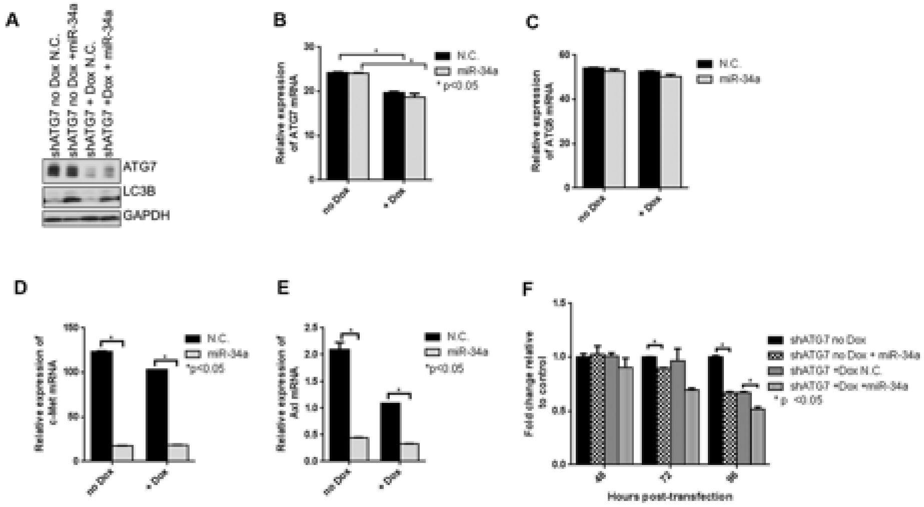

Figure S5: miR-34a induces autophagy independently of stable shATG7 knockdown. PC3 cells with shATG7 in a doxycycline (dox)-inducible vector were created. (**A**) Western blots for ATG7, LC3B and GAPDH in non-induced and dox-

induced cells with N.C. or miR-34a transfection. mRNA expression for (**B**) ATG7, (**C**) ATG5, (**D**) c-Met and (**E**) Axl. (**F**) Proliferation of cells at indicated time points in non-induced and Dox-induced cells with N.C. or miR-34a transfection. * denotes p value <0.05 as measured by student's t test.
